# Theoretical—Experimental Approach of Chitosan/Quaternized Chitosan Nanofibers’ Behavior in Wound Exudate Media

**DOI:** 10.3390/pharmaceutics15122722

**Published:** 2023-12-02

**Authors:** Bianca-Iustina Andreica, Alexandru Anisiei, Manuela-Maria Iftime, Razvan-Vasile Ababei, Lacramioara Ochiuz, Decebal Vasincu, Ingrid-Andrada Vasilache, Constantin Volovat, Diana Boboc, Vladimir Poroch, Lucian Eva, Maricel Agop, Dragos-Viorel Scripcariu, Simona Ruxandra Volovat

**Affiliations:** 1 “Petru Poni” Institute of Macromolecular Chemistry, Gr. Ghica Voda Alley, 41A, 700487 Iasi, Romania; andreica.bianca@icmpp.ro (B.-I.A.); anisiei.alexandru@icmpp.ro (A.A.); ciobanum@icmpp.ro (M.-M.I.); 2 Laboratory of Applied Meteorology and Climatology, A Building, Physics, Research Center with Integrated Techniques for Atmospheric Aerosol Investigation in Romania, RECENT AIR, Alexandru Ioan Cuza University of Iasi, 11 Carol I, 700506 Iasi, Romania; razvan.ababei@uaic.ro; 3 Faculty of Pharmacy, “Grigore T. Popa” University of Medicine and Pharmacy, 700115 Iasi, Romania; lacramioara.ochiuz@umfiasi.ro; 4 Department of Biophysics, Faculty of Dental Medicine, “Grigore T. Popa” University of Medicine and Pharmacy, Iasi 700115, Romania; decebal.vasincu@umfiasi.ro; 5 Department of Obstetrics and Gynecology, “Grigore T. Popa” University of Medicine and Pharmacy, 700115 Iasi, Romania; 6 Department of Medical Oncology-Radiotherapy, “Grigore T. Popa” University of Medicine and Pharmacy, 16 University Str., 700115 Iasi, Romania; dianaiboboc@gmail.com (D.B.); simonavolovat@gmail.com (S.R.V.); 7 Faculty of Medicine, “Grigore T. Popa” University of Medicine and Pharmacy Iasi, 700115 Iasi, Romania; vladimir.poroch@umfiasi.ro; 8 “Prof. Dr. Nicolae Oblu” Emergency Clinical Hospital, 2 Ateneului Street, 700309 Iasi, Romania; lucian_eva@yahoo.com; 9 Department of Physics, “Gheorghe Asachi” Technical University of Iasi, 700050 Iasi, Romania; magop@tuiasi.ro; 10 Romanian Scientists Academy, 050094 Bucharest, Romania; 11 Department of Surgery, “Grigore T. Popa” University of Medicine and Pharmacy, 16 University Str., 700115 Iasi, Romania; dscripcariu@gmail.com

**Keywords:** chitosan fibers, biomaterials, engineering nanomaterials, composite nanomaterials, enzymatic degradation

## Abstract

This study aimed to investigate the behavior of chitosan/quaternized chitosan fibers in media mimicking wound exudates to understand their capacities as wound dressing. Fiber analysis of the fibers using dynamic vapor sorption proved their ability to adsorb moisture up to 60% and then to desorb it as a function of humidity, indicating their outstanding breathability. Dissolution analyses showed that quaternized chitosan leached from the fibers in water and PBS, whereas only small portions of chitosan were solubilized in water. In media containing lysozyme, the fibers degraded with a rate determined by their composition and pH, reaching a mass loss of up to 47% in media of physiologic pH. Notably, in media mimicking the wound exudate during healing, they adsorbed moisture even when their mass loss due to biodegradation was high, whereas they were completely degraded in the media of normal tissues, indicating bioabsorbable dressing capacities. A mathematical model was constructed, which characterized the degradation rate and morphology changes of chitosan/quaternized chitosan fibers through analyses of dynamics in scale space, using the Theory of Scale Relativity. The model was validated using experimental data, making it possible to generalize it to the degradation of other biopolymeric systems that address wound healing.

## 1. Introduction

**Figure 1:**
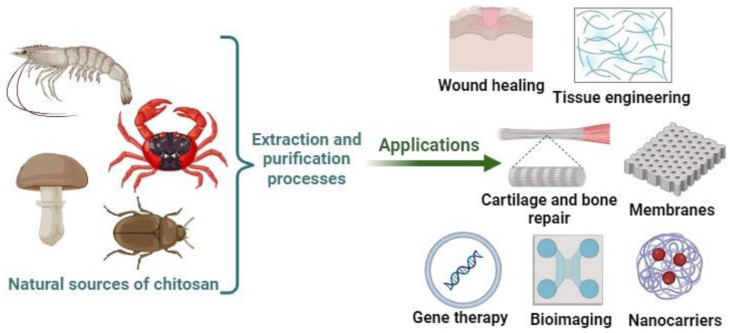
Biomedical applications of chitosan (created with BioRender.com, accessed on 25 September 2023).

Wounds impact the life quality of nearly 2.5% of the population worldwide, with an estimated cost 2–4% of the total healthcare expenses directed toward wounds’ treatment in Europe [1]. This situation is expected to worsen owing to the continuous increase in the obesity rate and related illnesses, such as diabetes, vascular deficits, hypertension, and chronic kidney diseases, all comorbidities that increase the incidence of chronic wounds. However, nosocomial infections threaten the normal healing of wounds, especially in low- and middle- income countries with deficient healthcare systems [2]. Therefore, it is necessary to develop new and efficient wound dressings. Many types of materials have been developed for this purpose, including foams, films, hydrocolloids, hydrofibers, hydrogels, and sprays [3,4,5,6], based on synthetic or naturally originating polymers [7,8,9]. Some of these, based on nylon, calcium alginate, hyaluronic acid, collagen, poly(vinyl alcohol) or silk, have passed the barrier from research to application [10,11], but they address only a few types of wounds and have a high cost. Traditional gauze bandages are currently in use in most hospitals, their disadvantage being the adhesion to the wound surface, making mechanical debridement necessary, damaging new epithelial tissue, and having repercussions on healing. A solution that can potentially overcome this disadvantage is the development of biodegradable dressings based on water-soluble polymers including gelatin [12], carboxymethyl chitosan [13], or polyethylene glycol [14], and others [15,16]. Among the materials of natural origin for wound dressings, chitosan stands out because of its favorable properties for healing. These properties include biocompatibility, bioadhesiveness, lack of toxic and allergenic effects, and hemostatic and antimicrobial activities [17,18,19]. A considerable amount of work was focused on developing wound dressings based on chitosan, such as hydrogels, membranes, sponges, and fibers, all of which have reported promising results [20,21,22]. Among these, chitosan-based nanofibers appear to be the most favorable because of their similarities to the natural extracellular matrix, good absorbency, semipermeability, and exceptional conformability [17,23,24,25,26,27]. They can be prepared via electrospinning, a simple, versatile, and green technology, which also has the advantage of leading to lightweight materials that have a high active area and are suitable for wound dressings [28,29,30,31]. Moreover, chitosan proved immunogenicity [32,33,34], its chemical structure bearing amine units confers solubility [35,36,37,38,39,40,41,42], and its degradation products are non-toxic and promote the growth of granulation tissue (Figure 1) [43,44].

The question is, how does the biodegradability of chitosan match the healing rate of wounds? Can it be used to design biodegradable materials that can overcome the difficult issue of debridement? Although many studies have highlighted that chitosan is enzymatically biodegraded in biological fluids, less attention has been paid to the relationship between the variable pH of wound exudate and chitosan’s biodegradation rate [45,46]. Moreover, no information regarding the influence of the biodegradation rate on the ability of dressings to adsorb wound exudates has been provided. The dynamicity and complexity of wound healing is due to the continuous processes of cellular structure and tissue layer replacement. It occurs in four phases: hemostasis, inflammation, proliferation, and remodeling, each of which is characterized by a different exudate pH [47]. Therefore, the rational question is: how does the pH of the exudate affect the biodegradation of chitosan-based materials and how does biodegradation affect the swelling ability of the fibers?

To answer this question, we used a series of chitosan/quaternized chitosan nanofibers, which are promising materials for the development of wound dressings [45], and analyzed their enzymatic biodegradation as a pH variable and its influence on the swelling ability. To this end, composite chitosan/quaternized chitosan nanofibers were prepared via electrospinning a ternary chitosan/quaternized chitosan polyethylene oxide (PEO) solution, followed by complete PEO removal. Their biodegradation was investigated by solubility and enzymatic degradation tests at different pH values corresponding to different wound healing stages. Furthermore, we constructed a new mathematical model that explains both the morphologies and specific degradation mechanisms using the Theory of Scale Relativity (TSR) in scale space.

## 2. Theoretical Model

A mathematical model for fibers’ biodegradation was constructed based on the idea that biodegradation can be viewed as a release process for material parts, similar to a drug release process. Standard models employed to depict the complex dynamics of polymer drugs are based on empirical functions, such as the Higuchi, Peppas, and Weibull laws [48]. All of these models partially describe the delivery dynamics, morphologies, and mechanisms of release. In our case, we must explain the following steps: swelling of the fibers, quaternized chitosan leaching involving chitosan chains, and breaking of chitosan chains under lysozyme effects. Recently, we proposed a unitary model [49,50] to characterize the complex dynamics of drug delivery derived from TSR [51,52,53]. Regardless of the type of model in the discussion, the fundamental hypothesis is as follows. Supposing that any bi-component polymer system is structurally and functionally assimilated to a fractal object, its dynamics can be described through the motions of any physical system entity, dependent on the chosen scale resolution, on continuous and non-differentiable curves (fractal curves).

These considerations imply that, in the characterization of any bi-component polymeric system dynamics, instead of “working” with a single variable (regardless of its nature, including velocity and density) described by a strict non-differentiable function, it is possible to “work” only with approximations of this mathematical function, obtained by averaging them on different scale resolutions. Consequently, any variable intended to describe any bi-component polymeric system dynamics will perform as the limit of a family of mathematical functions, which is non-differentiable for null-scale resolutions, and differentiable otherwise. In other words, from a mathematical point of view, these variables can be explained through fractal functions, that is, functions dependent not only on the spatial and temporal coordinates but also on the scale resolution.

All of the above specifies that any description of the bi-component polymeric system dynamics requires simultaneous dynamic descriptions in the framework of the Scale Relativity Theory on two manifolds: one on the usual space and the other on the scale space. In this way, biodegradation curves within the usual space can be obtained, whereas, in the scale space, we can determine both the morphologies and biodegradation mechanisms by analyzing the dynamics. In the scale space, any bi-component polymeric system dynamics can be described by means of two fundamental variables: the logarithms of the resolutions and the scale time.

In this context, because the scale space is generalized to a non-differentiable and fractal geometry, various elements of the new description can also be used [51]:

Infinity of trajectories, leading to the introduction of a scale velocity field

(1) $$V=V(\ln\;L\left(\tau \right),\tau )$$

in the form

(2) $$V=\frac{d\ln\;L}{dL}$$

where L is the non-differential space-scale coordinate, and τ is the time scale coordinate; breaking down the derivative of the fractal space scale coordinates into two components: a “classical part” and a “fractal part”, both governed by a stochastic variable, related to the usual space, can be written as follows:

(3) $$\langle d\xi_{s}^{2}\rangle =2D_{s}d\tau$$

In Equation (3), $D_{s}$ is a constant coefficient assigned to fractal/nonfractal transition in the scales space, and $\xi_{s}$ is the fractal term of the differential spatial coordinate in the scales space;

(i). Introduction of the two-valuedness of this derivative because of the symmetry breaking of the reflection invariance under the exchange dτ↔−dτ, giving a rise of a complex scale velocity $\tilde{V}$ based on this two-valuedness;
(ii). The construction of a new total covariant derivative with respect to the τ, which can be written as:
  (4) $$\frac{\hat{d}}{d\tau }=\frac{\partial }{\partial \tau }+\tilde{V}\frac{\partial }{\partial \ln\;L}-iD_{s}\frac{\partial^{2}}{{\left(\partial \ln\;L\right)}^{2}}$$
(iii). The introduction of a wave function as a re-expression of the action, which is now complex:
  (5) $$\Psi_{s}\left(\ln\;L\right)=exp\left(iS_{s}/2D_{s}\right)$$

In Equation (5), $\Psi_{s}$ represents the state function in scale space and $S_{s}$ is the action in scale space.

(iv). Transformation and integration of the free Newtonian scale-dynamics equation
  (6) $$\frac{d^{2}\ln\;L}{{d\tau }^{2}}=0$$

Under the form of a Schrödinger-type equation now acting on scale variables:

(7) $$D_{s}^{2}\frac{\partial^{2}\Psi_{s}}{{\left(\partial \ln\;L\right)}^{2}}+iD_{s}\frac{\partial \Psi_{s}}{\partial \tau }=0$$

### Dynamics in Scales Space through Riccati Gauge Type

Similar to Equation (7), Schrödinger equation admits an SL(2,R)-type group for two variables with three parameters. Working in the variables $\left(\ln\alpha ,\tau \right)$, the finite equations of this group are given by the following transformations:

(8a) $$\tau \to \frac{\alpha \tau +\beta }{\gamma \tau +\delta };$$

(8b) $$\ln\;L\to \frac{\ln\;L}{\gamma \tau +\delta }$$

These transformations represent the manifestation of the SL(2,R) framework using the variables (ln*α*, *τ*), featuring three crucial parameters. In this context, any vector within the tangent space of SL(2,R) can be expressed as a linear combination of three fundamental vectors, which are the infinitesimal generators.

(9) $$X_{1}=\frac{\partial }{\partial \tau },\;\;\;X_{2}=\tau \frac{\partial }{\partial \tau }+\frac{\ln L}{2}\frac{\partial }{\partial \ln L},\;\;\;X_{3}=\tau^{2}\frac{\partial }{\partial \tau }+\tau \frac{\partial }{\partial \ln L}$$

Satisfying the basic structure equations:

(10) $$\left[X_{1},X_{2}\right]=X_{1},\;\;\;\left[X_{2},X_{3}\right]=X_{3},\;\;\;\left[X_{3},X_{3}\right]=-2X_{2}$$

Which is used as the standard commutation relation for this type of algebraic structure throughout the present work.

Now, consider Equation (8a), which represents the homographic action of the generic matrix denoted by $\hat{M}$:

(11) $$\hat{M}=\left(\begin{array}{cc} \alpha & \beta \\ \gamma & \delta \end{array}\right)$$

The problem that we want to solve is to determine the relationship between the set of matrices and the set of values of τ for which τ remains constant. From a geometrical point of view, this means finding the set of points $\left(\alpha ,\beta ,\gamma ,\delta \right)$ that uniquely correspond to the values parameter τ.

Using Equation (8a), our problem is solved by a Riccati differential equation, which is obtained as a consequence of the constancy of τ:dτ=0:

(12) $$d\tau +\omega_{1}\tau^{2}+\omega_{2}\tau +\omega_{3}=0$$

where we use the notations:

(13a) $$\omega_{1}=\frac{\gamma d\alpha -\alpha d\gamma }{\alpha \delta -\beta \gamma }$$

(13b) $$\omega_{2}=\frac{\delta d\alpha -\alpha d\delta +\gamma d\beta -\beta d\gamma }{\alpha \delta -\beta \gamma }$$

(13c) $$\omega_{3}=\frac{\delta d\beta -\beta d\delta }{\alpha \delta -\beta \gamma }$$

The three differential 1-forms in Equations (13a)–(13c) constitute a co-frame at any point in absolute space. This co-frame allows us to translate the geometric properties of absolute space into algebraic properties related to the differential Equation (12).

The simplest of these properties refers to the motion on geodesics of the metric $ds^{2}=\frac{1}{4}(\omega_{2}^{2}-4\omega_{2}\omega_{2})$. In this case the 1-forms $\omega_{1}$, $\omega_{2}$, and $\omega_{3}$ are exact differentials in the same parameter as the length of the arc of the geodesic, let’s assume. Along these geodesics, Equation (12) becomes an ordinary Riccati-type differential equation:

(14) $$\frac{d\tau }{ds}=a_{1}\tau^{2}+2a_{2}\tau +a_{3}$$

Here, parameters $a_{1,2,3}$ are constants that characterize a certain geodesic of the family. Therefore, for obvious physical reasons, it is important to find the most general solution to Equation (14). Hence, we consider that:

(15) $$\tau_{0}\equiv -\frac{a_{2}}{a_{1}}+i\frac{\Omega }{a_{1}},\tau_{0}^{*}\equiv -\frac{a_{2}}{a_{1}}-i\frac{\Omega }{a_{1}};\Omega^{2}=a_{3}a_{1}-{a_{2}}^{2}$$

As the root of the quadratic polynomial on the right-hand side of Equation (14). Therefore, we first perform a homographic transformation as follows:

(16) $$z=\frac{\tau -\tau_{0}}{\tau -\tau_{0}^{*}}$$

It can be seen through direct calculations that z is a solution to the linear and homogeneous equation of the first order:

(17) $$\dot{z}=2i\Omega z∴z(\tau )=z(0)e^{2i\Omega \tau }$$

Therefore, if we conveniently express the initial condition z(0), we can obtain the general solution of Equation (14) by simply inverting the transformation in Equation (16), with the following result:

(18) $$\tau =\frac{\tau_{0}+re^{2i\Omega \left(\tau -\tau_{r}\right)}\tau_{0}^{*}}{1+re^{2i\Omega \left(\tau -\tau_{r}\right)}}$$

where r and $\tau_{r}$ are real constants that characterize the solution. Using Equation (15), this solution can be written in real terms i.e.,:

(19) $$z=-\frac{a_{2}}{a_{1}}+\frac{\Omega }{a_{1}}\left(\frac{2rsin\;\left[2\Omega \left(\tau -\tau_{r}\right)\right]}{1+r^{2}+2rcos\;\left[2\Omega \left(\tau -\tau_{r}\right)\right]}\left.+i\frac{1-r^{2}}{1+r^{2}+2rcos\;\left[2\Omega \left(\tau -\tau_{r}\right)\right]}\right)\right.$$

which highlights the frequency modulation of type. Moreover, the following notation is applied:

(20) r≡cothΦ

Equation (19) becomes:

(21) $$z=-\frac{a_{1}}{a_{2}}+\frac{\Omega }{a_{1}}h,\;\;\;\Omega =\surd {(a}_{1}a_{3}-a_{2}^{2})$$

In which h is given by:

(22) $$h=-i\frac{cosh\Phi -e^{-2i\Omega \left(\tau -\tau_{m}\right)}sinh\Phi }{cosh\Phi +e^{-2i\Omega \left(\tau -\tau_{m}\right)}sinh\Phi }$$

**Figure 2:**
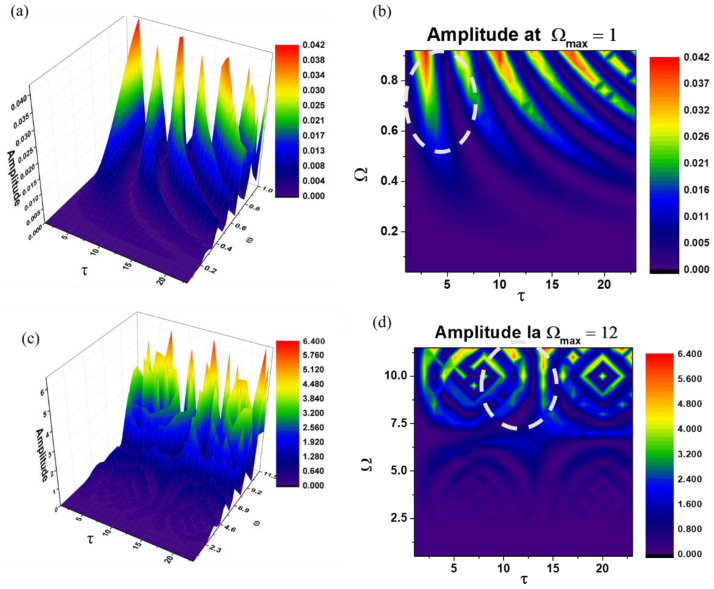
2D and 3D Plots of $Re\left(a_{1}z+a_{2}\right)$ as a function of Ω and τ. Graphs (**a**,**b**) illustrate the delivery channels for $\Omega_{max}$ = 1 and (**c**,**d**) show the degradation process of the polymer lattice under the lysosime effects for $\Omega_{max}$ = 12. The dashed line circle highlights a delivery channel in (**b**) and the degradation process in (**d**).

It should be noted that h can be obtained as a natural result of harmonic mapping from a typical Euclidian space to SL(2R) [32,33]. Figure 2a,b illustrates the dependencies of $Re\left(a_{1}z+a_{2}\right)$ on the dimensionless coordinates Ω and τ of Ω for the two values imposed by means of $\Omega_{max}=1$. These graphs show the morphologies of the leaching channels during biodegradation, as shown in Figure 2c,d.

**Figure 3:**
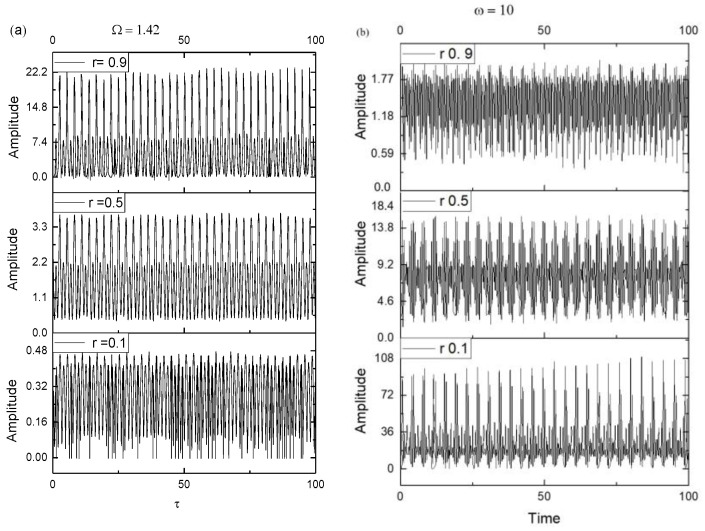
Plot of $Re\left(a_{1}z+a_{2}\right)$ dependence as a function of τ for r = 0.1, 0.5, and 0.9. Graph (**a**) shows a double period regime which Ω = 1.0, and graph (**b**) shows the intermittency regime where Ω = 10.

The correspondence through Equations (21) and (22) with harmonic mappings allows us to reproduce the mechanisms that can be associated with the imposed functionalities, either through the leaching mechanism shown in Figure 3a or through the degradable lattice shown in Figure 3b. This reveals double-period regimes specific to the functionalities imposed through leaching channels and intermittency that describe the biodegradable lattices.

## 3. Experimental Methods

### 3.1. Materials

Low-molecular-weight chitosan (126 kDa determined by viscosity measurements, degree of acetylation of 3% determined from the ^1^H-NMR spectrum) was prepared by alkaline hydrolysis of commercial chitosan (197 kDa, DA = 16%, determined as described above) purchased from Sigma Aldrich. *N*-(2-hydroxy)propyl-3-trimethyl ammonium chitosan chloride (Q) with a quaternization degree (DQ) of 54.4% was synthetized from commercial chitosan, following previously reported procedures [24,45,46], as briefly described below. Lysozyme from chicken egg white, lyophilized powder, protein ≥90%, ≥40,000 units/mg protein, PEO (1000 kDa), and all other reagents and solvents were purchased from Sigma–Aldrich and used as received.

### 3.2. Synthesis of N-(2-hydroxy)propyl-3-trimethyl Ammonium Chitosan Chloride (Q)

3 g chitosan was added into a reaction vessel containing 6 mL bidistilled water heated at 85 °C and gently magnetic-stirred for 2 h. Then, 6.6 g of glycidyltrimethylammonium chloride was slowly poured into the reaction vessel as portions of 2.2 mL added at each 1 h, and the reaction mixture was kept under stirring at 85 °C for 18 h, until it turned into a dark yellow, viscous liquid. At this point, 30 mL distilled water was added and the system was maintained for another 6 h to perform the reaction. Then, the reaction mixture was poured into cold acetone, kept at 0 °C for 18 h, and centrifuged at 4000 rot/min. The obtained solid was washed twice with cold acetone, allowed to dry, and then was dissolved in water, filtered, and lyophilized to give a white solid, which was characterized by FTIR spectroscopy and conductometric titration, which confirmed the obtaining of Q with a quaternization degree of 54.4%.

### 3.3. Preparation of the Chitosan/Quaternized Chitosan Fibers

**Figure 4:**
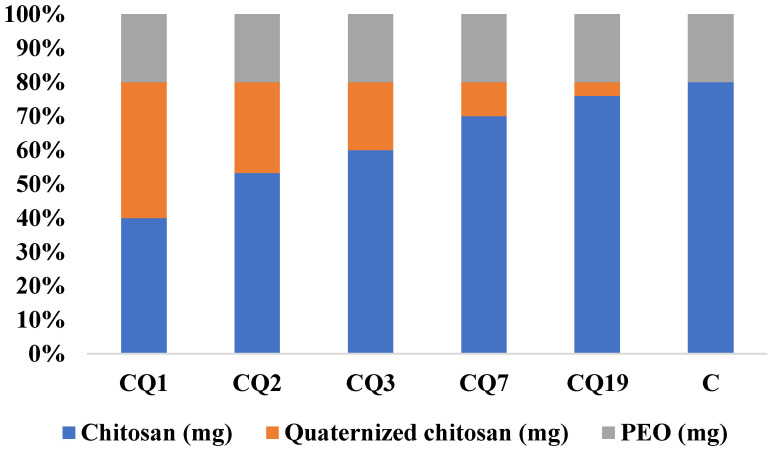
Composition of fibers before PEO removal and their codes.

Composite chitosan/quaternized chitosan fibers were produced according to a previously reported procedure [45]. Briefly, a 2.5% ternary solution was prepared from chitosan, quaternized chitosan, and PEO dissolved in 75% acetic acid, at room temperature, keeping the percent of PEO constant while varying the chitosan/quaternized chitosan ratio (Figure 4); this was then subjected to electrospinning (Tong Li Tech Tl-Pro-Bm equipment). The following parameters were used: distance between tip and collector was 12 cm, speed of rotary drum collector was 800 rpm, flow rate was 0.2 or 0.3 mL/h, room temperature was 25 °C, and the voltage varied from 8 to 10 kV to obtain a Taylor cone. An infrared lamp was placed at 20 cm from the collector in order to aid the faster drying of the fibers. The compositions of the fibers and their codes are shown in Figure 4. PEO was removed by washing with ethanol, and the fibers were dried under vacuum.

### 3.4. Methods

The fibers’ composition was confirmed using a Fourier-Transformed Infrared (FTIR) Spectrophotometer (Bruker Spectrophotometer, VERTEX 70, Billerica, MA, USA) operating in the 4000–600 cm^−1^ range, 32 scans, 4 cm^−1^ resolution. The spectra were recorded on pieces of nanofibers (10 mm, approx. 1.5 mg) placed between two KBr tablets, and they were further processed with OPUS 6.5 software. Furthermore, to investigate the successful removal of PEO, thermogravimetrical curves were recorded with a Discovery TGA 5500 equipment (TA Instruments) on previously dried nanofiber samples of 2 ± 0.5 mg each, placed into 100 µL platinum pans at a heating rate of 10 °C/min, from 26 °C to 600 °C.

Dissolution of the fibers was monitored in water and PBS (pH 7.4). Four milligrams of each sample was immersed in 4 mL media and kept in an incubator (Cryste Puricell 80) at 37 °C, under gentle shaking (25 rpm). After 24 h, the samples were removed, washed with distilled water to remove salts from the PBS, lyophilized, and subjected to weighing and microscopic analyses [45]. The mass loss was determined using Equation (23).

W_loss_ (%) = [(W_0_ − W_t_)/W_0_] × 100 (23)

where W_0_ and W_t_ represent the masses of the fibers in the initial state and after a certain time, respectively.

**Figure 5:**
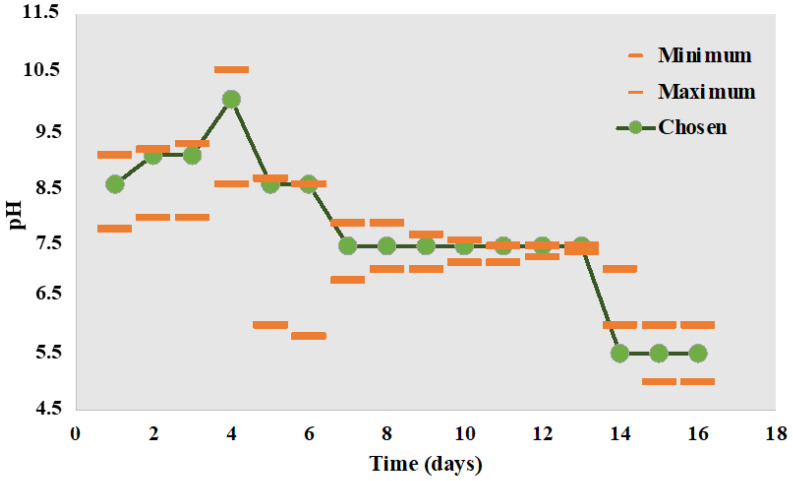
Graphical representation of the pH at different moments during the biodegradation experiment, adapted to fit the pH of wound exudate over the wound healing; (orange marks show the maximum and minimum values of pH determined from clinical trials; green marks represent the pH values chosen for this study) [54].

The nanofibers’ biodegradation was assessed in vitro, in a lysozyme solution (376 U/mL) prepared in PBS (pH 7.4). Pieces of nanofibers (4 ± 0.2 mg) were introduced in vials with 4 mL media and incubated into a Cryste Puricell 80 incubator, at 37 °C and 25 rpm. At certain time points (1, 3, and 7 days), the samples were removed, washed with distilled water to remove salts, dried for 24 h, and weighed. During the experiment, the medium was refreshed every three days to avoid inactivation of the enzyme. Mass loss was determined in a manner similar to that of the dissolution experiment (Equation (23)). To simulate the behavior of the fiber mats in contact with a wound, an experiment in which the pH of the media was progressively changed to fit the evolution of the exudate pH during the healing period was performed. Pieces of fibers were immersed in lysozyme media at pH = 8.5, and then, at certain moments, the media was replaced with one of the different pH values (Figure 5). A total of 21 fiber pieces were initially immersed in lysozyme media, and at each moment of media replacement, 3 of them were taken, washed, dried, and weighed.

The swelling behavior of the nanofibers was investigated in water, PBS (pH 7.4), and lysozyme media in PBS (376 U/mL) to mimic the wound exudate. Pieces of nanofibers of 4 ± 0.4 mg each were placed in previously weighed vials and 5 mL of media were added. At certain times, the media was removed (cleaning the excess on the vial walls with filter paper), and the vials were weighed. Furthermore, the data were used to calculate mass equilibrium swelling using Equation (24).

MES (g/g) = (Wt − W_0_)/W_0_ (24)

where W_0_ is the initial mass and Wt is the mass of the nanofibers at different weighing times.

The behaviour of the samples in media of variable humidity was assessed using a fully automated gravimetric analyzer (IGAsorp by Hiden Analytical equipment) at 25 °C, with relative humidity (RH) between 0 and 90%, and an increase of 10% humidity steps, following a standard protocol [25]. The morphology of the fibers before and after degradation was monitored using images acquired with a Scanning Electron Microscope (SEM) Verios G4 UC (Thermo Fisher Scientific, Waltham, MA, USA) and a Polarized Optical Microscope (POM) Zeiss Axio Imager M2. The average fibers’ diameter was determined with the ImageJ software 1.53p (ImageJ bundled with 64-bit Java 1.8.0_172).

## 4. Results and Discussions

**Figure 6:**
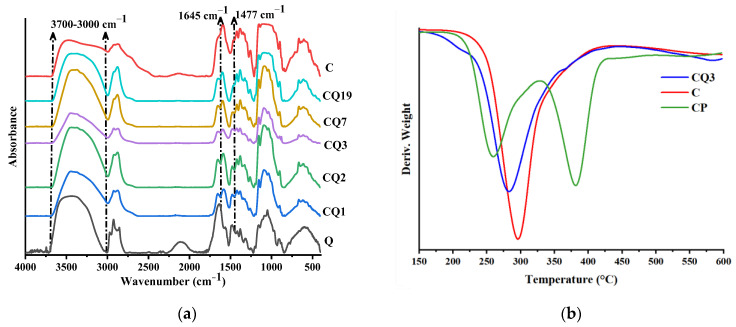
Confirmation of the fibers’ composition by (**a**) FTIR spectra, showing the characteristic vibration bands of chitosan and quaternized chitosan and (**b**) TGA derivative curves, showing the absence of degradation characteristic to PEO (CP stands for chitosan/PEO fibers).

A series of binary chitosan/quaternized chitosan nanofibers were prepared by electrospinning ternary blend solutions using PEO as a sacrificial component, which could be easily removed by selective washing with ethanol [24,25,45]. The codes of the samples were attributed, taking into consideration their composition, by abbreviating the chitosan with C and quaternized chitosan with Q, and using a number to express their ratio into nanofibers i.e., fibers with the mass ratio of chitosan/quaternized chitosan of 3/1 were coded CQ3, and so on (Figure 4). The fiber composition was confirmed by FTIR spectroscopy, which displayed the characteristic vibration band of quaternary ammonium salt around 1477 cm^−1^, along with other bands characteristic of chitosan: 3700–3000 cm^−1^ for vibration of -NH_2_, -OH groups, and H-bonds developed by them; 1645 cm^−1^ for stretching of C=O of *N*-acetyl groups (amide I); 1589 cm^−1^ for bending of amine units of chitosan; and 1100 cm^−1^ for vibration of -OH units (Figure 6a) [55]. Moreover, the lack of the decomposition step representative for PEO (300–400 °C) in the TGA curves demonstrated that the sacrificial component PEO was completely removed (Figure 6b).

**Figure 7:**
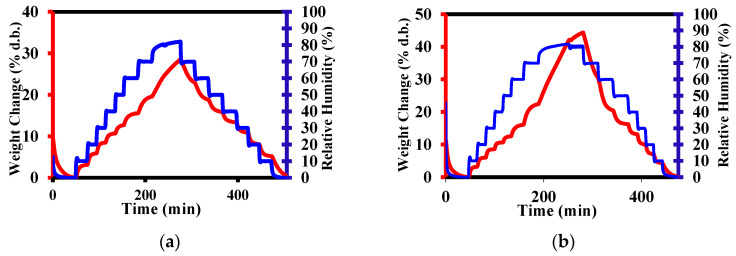

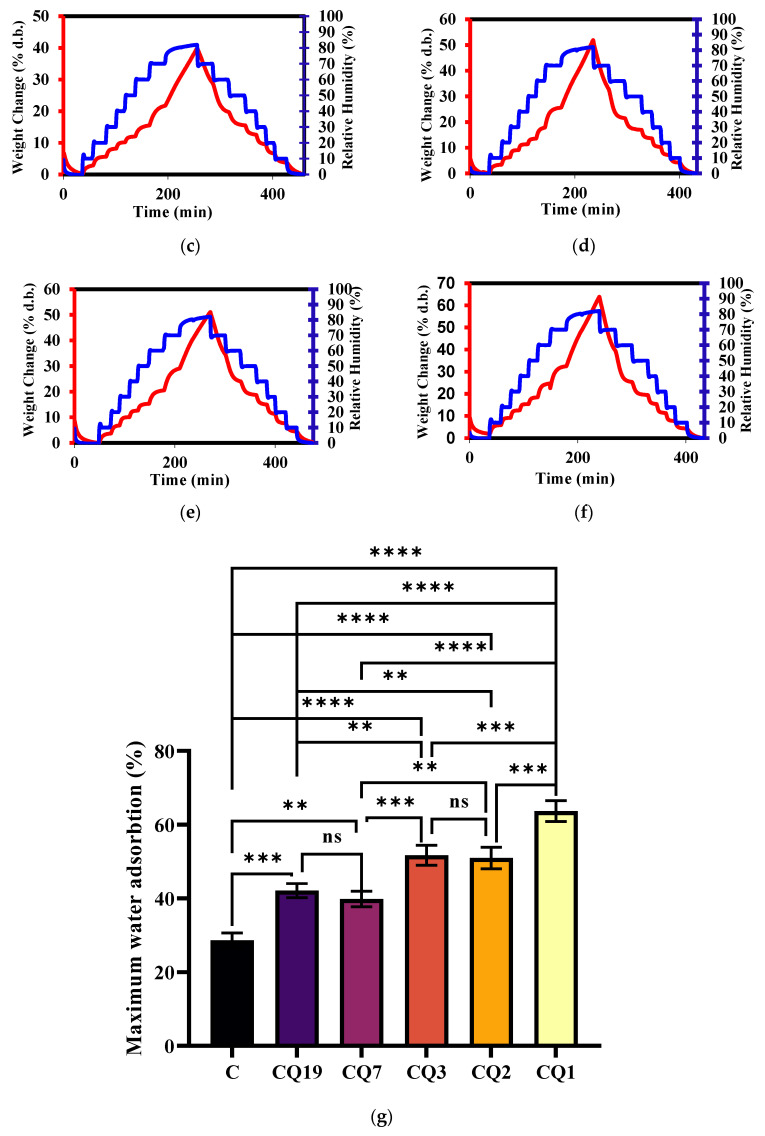
Dynamic vapor sorption of the (**a**) C, (**b**) CQ19, (**c**) CQ7, (**d**) CQ3, (**e**) CQ2, and (**f**) CQ1 samples over the increasing/decreasing humidity, and (**g**) statistical representation of the maximum vapor sorption of the samples (** *p* ˂ 0.01; *** *p* ˂ 0.001; **** *p* ˂ 0.0001, ns—not significant).

For a proper healing of wounds, dressings must have good breathability and absorb the exudate and donate it to the environment to prevent tissue maceration [56]. Therefore, the fibers’ capacity to adsorb and donate moisture was investigated by dynamic water vapor adsorption (Figure 7a–f). This experiment confirmed the improvement in liquid adsorption with increasing Q content, from 30 to 60%, even some minor, statistically insignificant alterations of this trend were noticed (Figure 7g), the most probably due to the influences of other factors, such as the shape of pores or distribution of the fiber diameters. Moreover, it was revealed that the fibers adsorb water when the humidity increases, and the water is desorbed when the humidity decreases, suggesting that when applied to exuding wounds, they will be able to adsorb exudate and donate it to a drier environment.

To understand the factors influencing the swelling behavior, processes that can occur in wet media, such as dissolution and biodegradation, were firstly investigated. The dissolution of the CQ fibers was also investigated in water and PBS. The solubility of the fibers increased with the Q content in both media, in accordance with their good water solubility [26].

**Figure 8:**
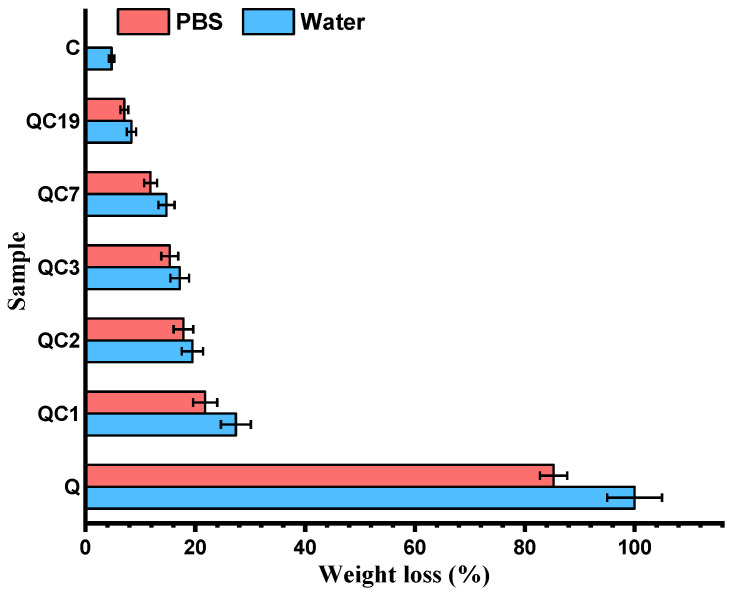
Solubility of fibers in water and PBS after 24 h, compared to the reference quaternized chitosan (coded Q).

The higher solubility of the fibers in water than in PBS was attributed to chitosan’s partial solubility in water and its lack of solubility in PBS (Figure 8).

**Figure 9:**
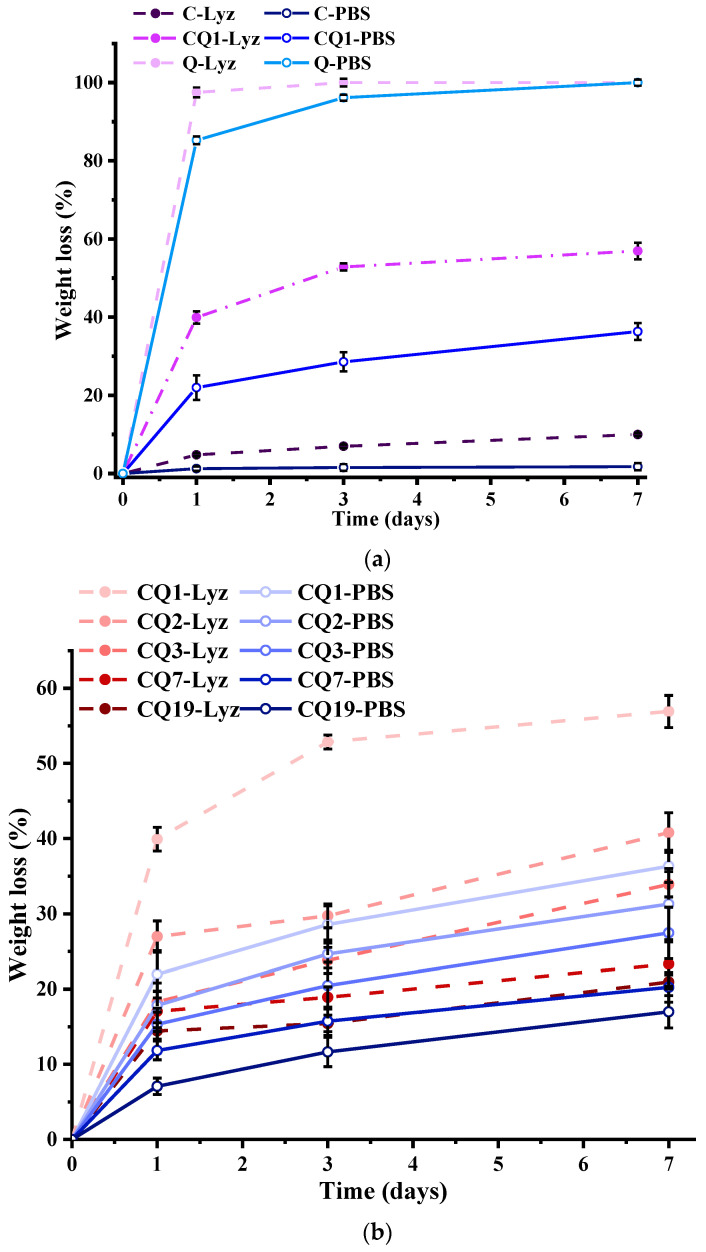

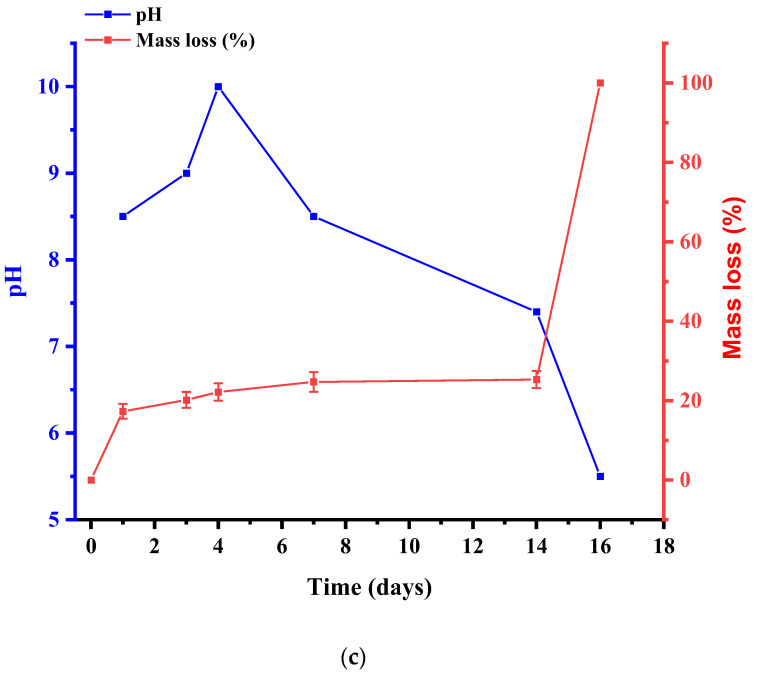
Graphical representation of the degradation of the CQ fibers in (**a**), (**b**) PBS and lysozyme in PBS (pH = 7.4) and (**c**) media of pH mimicking the pH evolution along the wound healing.

Furthermore, the biodegradation of the fibers in wet media was investigated. It is known that the biodegradation of chitosan-based materials is generally due to the degree of solubilization of chitosan and its enzymatic degradation. Moreover, the functionalization of chitosan with quaternary ammonium groups significantly improves its solubility [26,46,56,57,58]. Based on these data, the degradation of the nanofibers was investigated by considering their degree of solubilization and enzymatic degradation. Lysozyme, an enzyme found in biological fluids, including wound exudate, is known as the main factor contributing to chitosan degradation in the human body and was chosen for this purpose (Figure 9).

First, it was noticed that Q completely dissolved in lysozyme media after 24 h and in PBS after 7 days, whereas the pure chitosan fibers lost around 10% in lysozyme media after 7 days and barely around 2% in PBS. By combining chitosan and Q, the degradation of the fibers accelerated, the mass loss in lysozyme medium increasing from 15 to 55% along the Q content (Figure 7a,b). The degradation rate was higher in lysozyme media than in neat PBS, which is the rational outcome of summing the dissolution and enzymatic degradation. The degradation rate was higher in the first 24 h, when the process was governed by the dissolution of Q, and slower in the following days, when the process was governed by enzymatic degradation. Overall, the biodegradation of the fibers was improved in the presence of Q, and the degradation rate was controlled by the chitosan/Q ratio. It can be assumed that the leaching of the Q improved the access of the lysozyme into the nanofibers, improving the biodegradation rate. The faster degradation in the first 24 h, corresponding to the inflammatory stage of the wounds, is expected to be beneficial for healing, as the release of the antimicrobial Q in the exudate media should prevent infection [59]. Furthermore, the slow release of the degradation products of chitosan/Q in the days following cell proliferation should be beneficial for healing because they are known metabolites that stimulate the healing process [60].

Furthermore, mimicking the biodegradation process in media with a pH consistent with the pH of the exudate over the wound healing period revealed an excellent match, indicating that over the healing period, the chitosan/Q fibers should be bio-absorbed (Figure 9c). It can be observed that in media with a pH higher than 7, corresponding to the hemostasis and inflammation stages, the degradation grows progressively, and in media with lower pH, corresponding to the granulation and remodeling stages, the decomposition is abrupt, pointing to the total resorption of the fibers. This suggests that chitosan/Q fibers are suitable for other types of bioapplications, such as bioactive scaffolds for bone regeneration [61], fillings for dental pulp regenerative procedures [62], small-diameter vascular regeneration [63], rapidly dissolving transient electronics [64], and biostrips for electrochemical detection of analytes [65].

**Figure 10:**
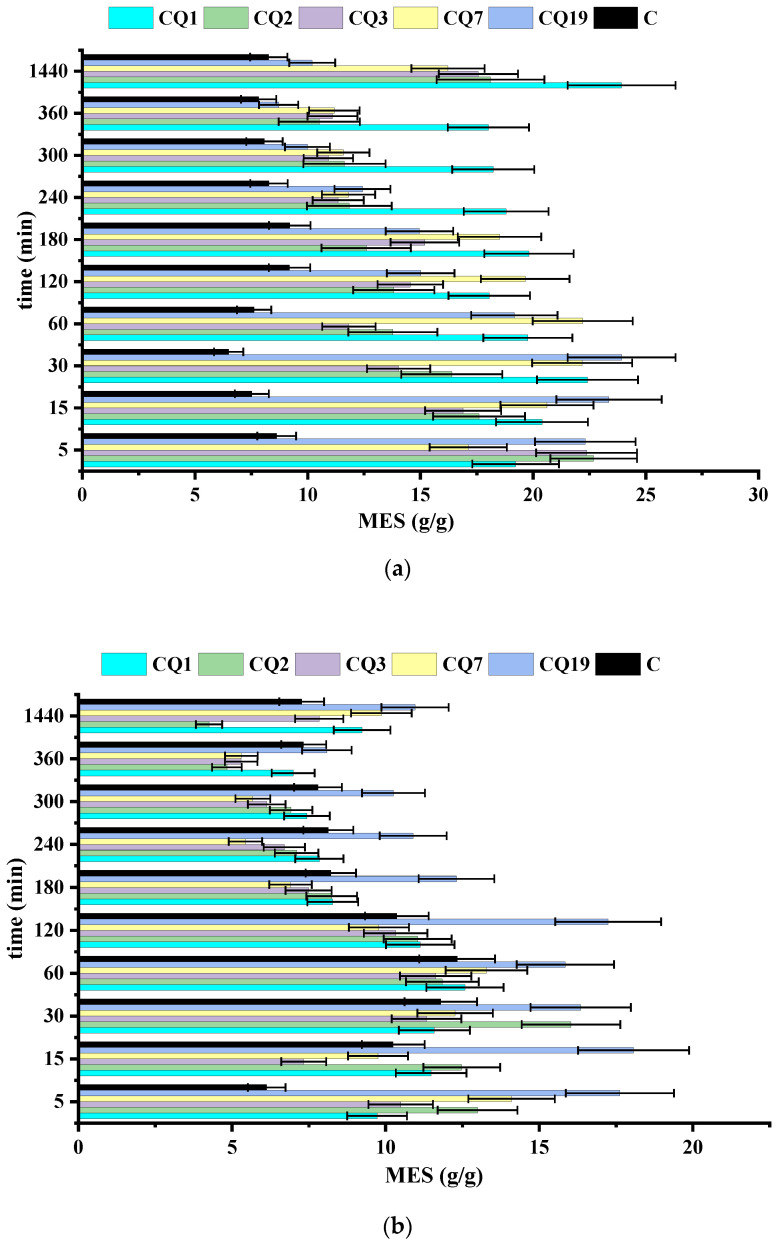

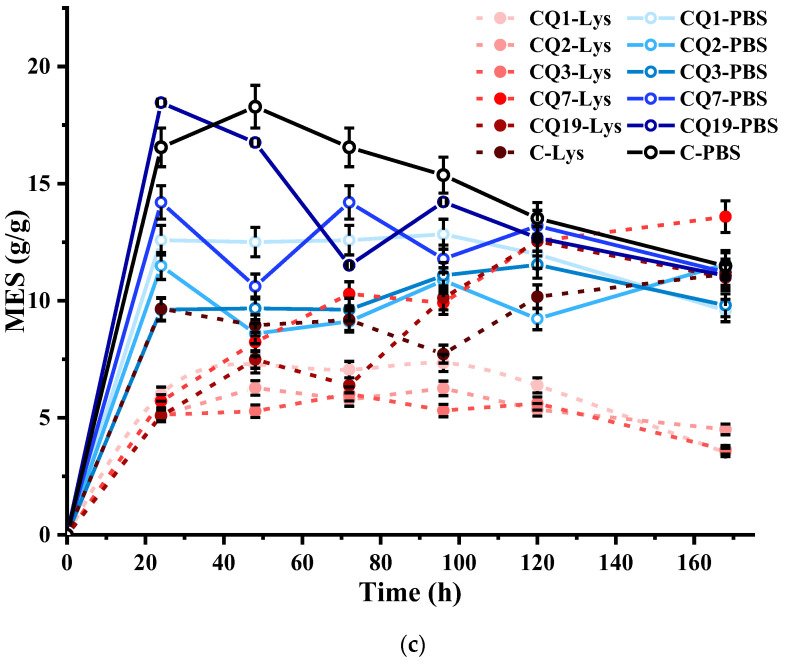
Swelling ratio of the fibers in (**a**) water and (**b**) PBS analyzed for 24 h and (**c**) lysozyme vs. PBS media, analyzed for 7 days.

The swelling of the fiber mats in water, PBS, and lysozyme media was investigated. The results are shown in Figure 10 and explained considering the influence of dissolution and biodegradation, as described above. In general, the presence of the hydrophilic Q considerably improved the fiber swelling ability in all cases, and swelling was a dynamic process over time (Figure 10a). Thus, in water, the rate of water uptake decreased slowly over 6 h and then increased, in line with the leaching of Q by dissolution, which probably created pores inside the fibers, favoring the swelling. The water retention ability increased as the amount of Q decreased, indicating that Q leaching was accompanied by fibers’ erosion.

In PBS, a decreasing swelling trend over time was observed (Figure 10b), in accordance with the lower ability of chitosan fibers to swell in basic media after Q leaching [51]. By adding lysozyme to PBS, the swelling ability decreased over time, reflecting the advanced erosion of the fibers due to the scission of chitosan chains and dissolution of the resulting oligomers [52,53]. In fibers with high Q content, a lower MES value than that in fibers with low Q content (CQ7 and CQ19) or no Q (C) was recorded. This was attributed to advanced fiber erosion and subsequent inferior capacity to accommodate solvent molecules. However, overall, the swelling ability was well-preserved, with a swelling degree of approximately 5 g/g even after 7 days. In contrast, the CQ7 and CQ19 fibers showed an improvement in swelling ability over time, reaching a swelling degree of approximately 12 g/g, which was higher than that in the absence of lysozyme (Figure 10c). These values indicate the fibers as suitable dressings for healing the dry-to-light exuding wounds and light-to-moderate exuding wounds, respectively [66].

Finally, considering the theoretical model that involves gauge transformation of Ricatti-type proved to work as scale resolution transformation, where gauge function becomes $\chi \left(r,t\right)=ln\rho$. In this context, the scale invariance leads to the following equation:

(25) $$\hat{D}\phi =\frac{\delta \phi }{\delta ln\rho }=const.$$

where $\hat{D}$ is the dilatation/contraction operator within the scale space. We impose a particular mathematical expression for ρ as:

(26) $$\rho =\tau^{b}exp\;(a\tau )$$

The solution of Equation (25) by choosing a convenient integration constant is given by Equation (27):

(27) ϕ=A·τ+B·lnτ

**Figure 11:**
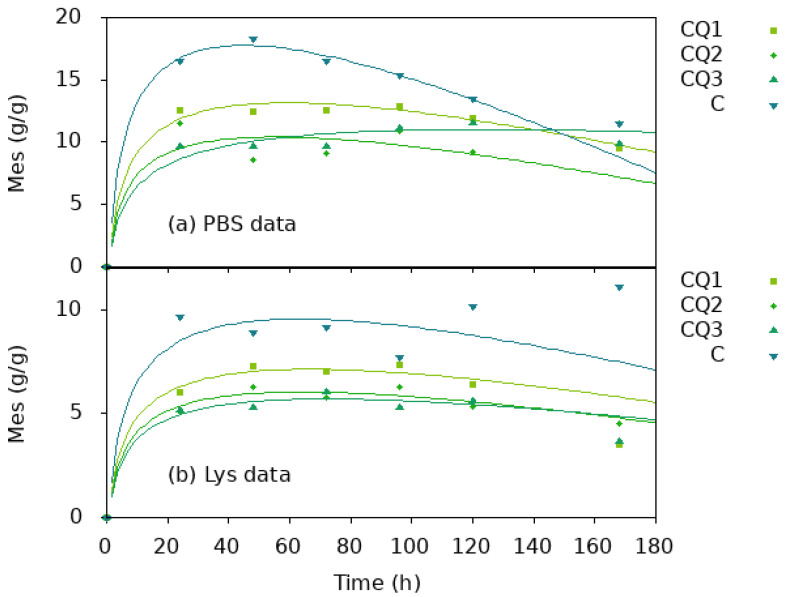
Fitting function for data presented in Figure 10c. The fitting function is: $Mes\;\left(\tau \right)=Mes_{0}\cdot \tau +\alpha \cdot ln\;(\tau )$.

Equation (27) represents the fitting of the experimental data shown in Figure 11, indicating the possible validation of our theoretical model. As can be seen, the data are fitting very well for the chitosan/quaternized chitosan fibers. For the neat chitosan nanofibers, the model fit well only over the first hour. This is probably due to the compactness of the fibers in the absence of Q leaching, which hindered the access of lysozyme into fibers and retarded the degradation.

**Figure 12:**
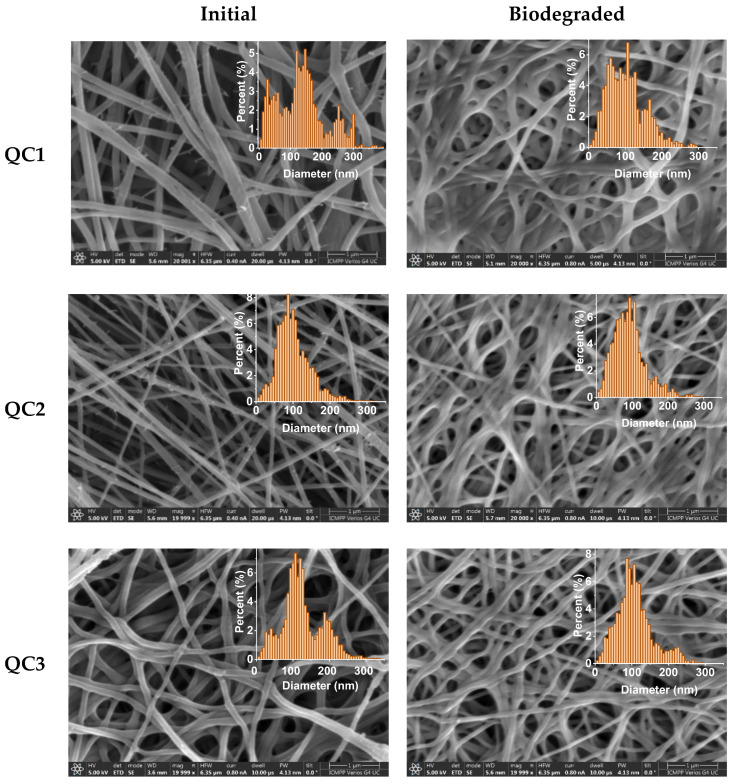

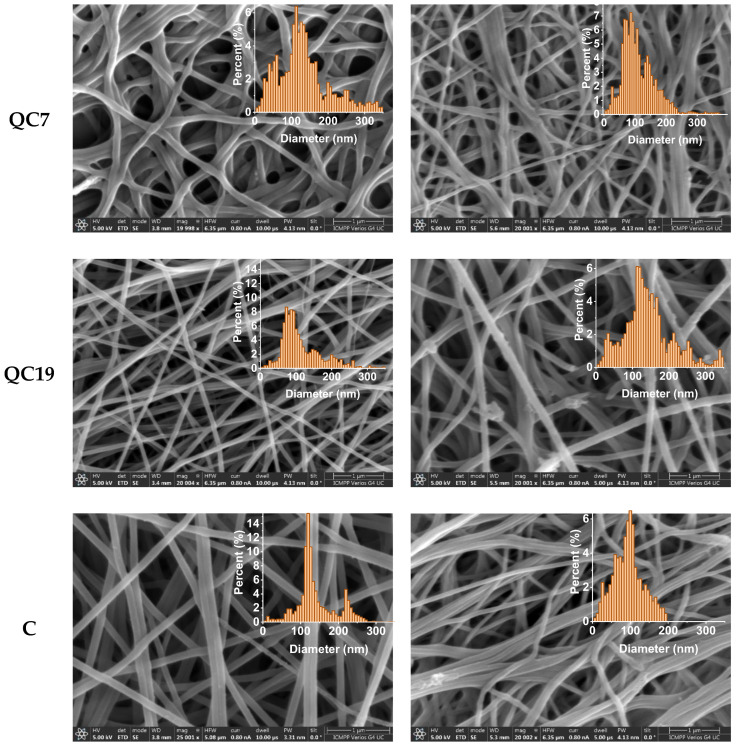
SEM micrographs of the fibers and corresponding histograms before (**left**) and after (**right**) the biodegradation experiment (scale bar: 1 µm, mag: 20,000 or 25,000, HV: 5 kV). The apparent dense aspect of the fibers may be the result of the reorganization of the fibers in lysozyme media, an aspect also observed for other chitosan-based nanofibers [24].

Analyzing swelling vs. biodegradation as concurrent processes, it can be concluded that the biodegradation impacts the swelling via two antagonistic processes: on the one hand, via the occurrence of pores inside the fibers, which are favorable for swelling, and on the other hand, via fiber erosion, which affects the integrity of the fibers and their ability to retain liquids. To determine the extent to which erosion affected swelling, the morphology of the fibers was monitored using SEM images acquired before and after fiber degradation (Figure 12, Table 1). Overall, after the biodegradation experiment, the fiber diameter significantly diminished. Moreover, some fibers stuck together, leading to an apparent increase in fiber diameter. Moreover, the analysis of the diameter histograms showed that the thin fibers almost vanished after biodegradation, and the diameter of the most common fibers decreased. These results indicate that biodegradation occurred mainly at the surface, leading to fiber erosion.

## 5. Conclusions

This study reports the behavior of a series of chitosan/quaternized chitosan fibers in media that mimics wound exudates. It was demonstrated that the swelling kinetics are closely correlated with the composition of the fibers, i.e., the ratio of the chitosan/Q and nature of the environment. Thus, in media mimicking the wound exudate, competition occurs between the leaching of Q and chitosan’s oligomers resulting from enzymatic degradation and erosion of the fibers. This competition resulted in pores forming inside the fibers, which was favorable for the retention of liquids, despite the significant fiber erosion. For the fibers with a Q content higher than 25%, the swelling degree preserved values higher than 5 g/g even after 7 days, whereas for those with lower Q content, the values were higher than 12 g/g, suggesting their propensity for application in dry-to-light exuding wounds and light-to-moderate exuding wounds, respectively. Moreover, the fibers proved excellent breathability, with the capacity to adsorb up to 60% water vapors and to donate them to a drier environment. This is an important finding for the application of fibers in wound healing. A mathematical model developed based on the Theory of Scale Relativity fit well with the swelling profile, validating the evolution of the fiber morphology as a consequence of the biodegradation process in the spatial/temporal/scale resolution interplay. Overall, this study demonstrated that chitosan/quaternized chitosan fibers are an outstanding choice for wound dressings, allowing the adaptation of the biodegradation rate to wound healing requirements.

## Figures and Tables

**Table 1:** Fibers’ mean diameter.

	Initial						After Biodegradation					
Code	QC1	QC2	QC3	QC7	QC19	C	QC1	QC2	QC3	QC7	QC19	C
Mean (nm)	120.54	91.92	112.69	117.12	86.68	121.01	91.76	83.41	98.16	96.48	131.38	91.49
SD (nm)	82.24	36.43	44.17	54.44	24.36	10.61	50.36	38.19	38.27	42.72	48.85	37.13
HM (nm)	146.67	86.67	106.67	113.33	73.33	120.00	106.67	93.33	86.67	86.67	113.33	100.00

Mean: The fibers’ mean diameter obtained by fitting a Gaussian Curve to the radius data; SD (Standard Deviation): The SD of the Gaussian; and HM (Histogram Mode): most common fiber diameter in the histogram.

## Data Availability

Data are contained within the article.
